# Anti-inflammatory Role of Galectin-8 During *Trypanosoma cruzi* Chronic Infection

**DOI:** 10.3389/fcimb.2020.00285

**Published:** 2020-07-02

**Authors:** Adriano Bertelli, Liliana M. Sanmarco, Carla A. Pascuale, Miriam Postan, Maria P. Aoki, María S. Leguizamón

**Affiliations:** ^1^Instituto de Investigaciones Biotecnológicas, Universidad Nacional de General San Martín, San Martín, Argentina; ^2^Consejo Naciona de Investigaciones Científicas y Tecnológicas, Buenos Aires, Argentina; ^3^Departamento de Bioquímica Clínica, Facultad de Ciencias Químicas, Universidad Nacional de Córdoba, Córdoba, Argentina; ^4^Centro de Investigaciones en Bioquímica Clínica e Inmunología, Córdoba, Argentina; ^5^Departamento de Investigación, Instituto Nacional de Parasitología “Dr. Mario Fatala Chabén, Buenos Aires, Argentina

**Keywords:** preaparesis, inflammation, neutrophils, fibrosis, Chagas disease

## Abstract

Galectins are animal lectins with high affinity for β-galactosides that drive the immune response through several mechanisms. In particular, the role of galectin-8 (Gal-8) in inflammation remains controversial. To analyze its role in a chronic inflammatory environment, we studied a murine model of *Trypanosoma cruzi* infection. The parasite induces alterations that lead to the development of chronic cardiomyopathy and/or megaviscera in 30% of infected patients. The strong cardiac inflammation along with fibrosis leads to cardiomyopathy, the most relevant consequence of Chagas disease. By analyzing infected wild-type (iWT) and Gal-8-deficient (iGal-8KO) C57BL/6J mice at the chronic phase (4–5 months post-infection), we observed that the lack of Gal-8 favored a generalized increase in heart, skeletal muscle, and liver inflammation associated with extensive fibrosis, unrelated to tissue parasite loads. Remarkably, increased frequencies of neutrophils and macrophages were observed within cardiac iGal-8KO tissue by flow cytometry. It has been proposed that Gal-8, as well as other galectins, induces the surface expression of the inner molecule phosphatidylserine on activated neutrophils, which serves as an “eat-me” signal for macrophages, favoring viable neutrophil removal and tissue injury protection, a process known as preaparesis. We found that the increased neutrophil rates could be associated with the absence of Gal-8-dependent preaparesis, leading to a diminished neutrophil-clearing capability in macrophages. Thus, our results support that Gal-8 exerts an anti-inflammatory role in chronic *T. cruzi* infection.

## Introduction

Galectins (Gals) bind β-galactosides *via* carbohydrate recognition domains and modulate immune cell responses through several mechanisms. Specifically, galectin-8 (Gal-8) has been involved in homeostatic and pathological processes. It regulates cytokine production, cellular adhesion, apoptosis, chemotaxis, endocytosis, differentiation, and migration in a wide range of cell types including immune cells (Elola et al., 2014). High concentrations of Gal-8 have been proposed to induce a strong T-cell proliferation even in the absence of the specific antigen, whereas low concentrations co-stimulate T-cells in the presence of antigen-presenting cells and their cognate antigen (Tribulatti et al., 2012). Gal-8 induces firm but reversible adhesion of peripheral neutrophils to endothelial cells (Nishi et al., 2003). Together with platelet activation (Romaniuk et al., 2010), these processes suggest a potential pro-inflammatory role for Gal-8. Other authors emphasize that Gal-8 exhibits anti-inflammatory effects on autoimmune diseases such as rheumatoid arthritis (Eshkar Sebban et al., 2007), experimental models of uveitis (Sampson et al., 2015), and encephalomyelitis (Pardo et al., 2017). With the use of an *in vitro* approach, Gal-8 was found to be involved in preaparesis induction, a cell removal mechanism that prevents local inflammation and systemic immune response activation. Cells undergoing preaparesis or apoptosis is signaled by expose phosphatidylserine (PS) as signals for phagocytosis, but preaparesis only removes viable neutrophils (Stowell et al., 2008).

These controversies on Gal-8 role in inflammatory processes led us to analyze its impact in a chronic inflammatory infectious disease, employing the *Trypanosoma cruzi* protozoan infection in a murine model.

Host–parasite interaction induces alterations that lead to the development of chronic megaviscera and/or cardiomyopathy in ~30% of infected patients, with the latter being the most frequent and severe. Chronic Chagas cardiomyopathy is a consequence of cardiac inflammation and fibrosis caused by local parasite persistence. These alterations are reproduced in mice, thus providing a suitable experimental model for Chagas disease cardiomyopathy. It is currently accepted that tissue parasite burden triggers the inflammatory response underlying the cardiac disorders that generate cardiomyopathy (Garcia et al., 2005; Marin-Neto et al., 2007; Weaver et al., 2019). The mechanisms involved, however, are not completely understood. After infection, *T. cruzi* invades endothelial cells, macrophages, fibroblasts, and dendritic cells but presents a particular tropism to cardiac cells. Cardiomyocyte infection triggers a complex process that leads to cardiac damage and hypertrophy, loss of network communication, proliferation of cardiac fibroblasts, and intense extracellular matrix (ECM) remodeling ending in cardiac insufficiency and death (Rassi et al., 2010). On its way to the target cells, the trypomastigote (infective stage) must leave the bloodstream and interact with the ECM (fibronectin, laminin, and galectins), which involves adhesion and migration events. Gal-8 may also be involved in these processes, as it is expressed in several tissues. Considering that the binding of recombinant human Gal-8 to trypomastigotes favors cellular adhesion (Pineda et al., 2014) and the parasite surface is covered by a heavily *O*-glycosylated mucin coat (Mucci et al., 2017), this galectin could also be involved in this interplay.

Using Gal-8-deficient mice, we observed that Gal-8 exerts an anti-inflammatory role during *T. cruzi* chronic infection. We also provide evidence that Gal-8 could induce neutrophil preaparesis *in vivo*.

## Methods

### Ethics Statement

The study was carried out in accordance with the Basel Declaration. Protocol (No. 10/2017) was approved by the Committee for Experimental Animal Care and Use of the Universidad Nacional de San Martín (UNSAM), following the recommendations of the *Guide for the Care and Use of Laboratory Animals* of the National Institutes of Health (NIH).

### Mice

Male C57BL/6J (B6) mice were from the colony established in our facilities from breeder pairs obtained from The Jackson Laboratory (Bar Harbor, ME, USA). Male mice deficient in Gal-8 *Lgals8* gene [B6; 129S5-*Lgals8Gt* (OST314218) Lex/Mmucd] were obtained from Mutant Mouse Resource & Research Centers (MMRRC; University of California, Davis, CA, USA) as heterozygotes. After 12 in-house backcrosses to B6, a homozygous Gal-8 knock-out colony with 95% of B6 genetic background was established, as assessed at The Jackson Laboratory Genotyping Resources. CF1 mice were bred from a colony obtained from Charles River Company. Mice were anesthetized with isoflurane before manipulation.

### Parasites and Experimental Infection

Male mice 10 to 16 weeks old were infected with 50,000 *Trypanosoma cruzi* Ac strain blood-derived trypomastigotes (DTU TcI) (Risso et al., 2004). This strain is maintained by serial passages in CF1 mice. Parasitemia values were recorded by counting trypomastigotes in a hemocytometer. The analysis of Gal-8 role in *T. cruzi* murine model was conducted at 4–5 months post-infection (mpi). Age-matched, normal B6, and Gal-8 knock-out mice were included as non-infected controls.

### Histopathology Assays

The skeletal muscle, heart, and liver were obtained from *T. cruzi*-infected mice and controls, fixed in 10% buffered formalin, and embedded in paraffin. Five-micron-thick sections were stained with hematoxylin and eosin and Masson's trichrome. A single blind microscopic evaluation of the tissue sections was performed on pre-coded slides and examined using an Olympus DP71 light microscope. Skeletal muscle and heart inflammation was evaluated with respect to both distribution (focal, confluent, or diffuse) and extent of inflammatory cells as previously described (Tarleton et al., 1994; Martin et al., 2007). Briefly, tissues were scored 1+ for a single inflammatory focus; 2+ for multiple, non-confluent foci of inflammatory cell infiltrates; 3+ for multifocal confluent inflammation; and 4+ for diffuse inflammation extended through the section. Mean values of two skeletal muscle sections were used to obtain an inflammatory score. Heart inflammation was evaluated separately in the left and right atrial and ventricular walls and septum, and the mean of the inflammation scores obtained in the different areas is used to determine the inflammatory index. Liver involvement was evaluated according to the distribution of inflammatory cells in the tissue, as follows: 1+ for multiple, non-confluent foci of inflammatory cells; 2+ for areas of focal and diffuse inflammation; and 3 + for diffuse inflammation extended throughout the section. Heart fibrosis was scored as 1+ for focal, mild augmentation of the normal interstitial connective tissue; 2+ for multiple areas of interstitial connective tissue augmentation surrounding groups or individual fibers; and 3+ for intramyocardial scars with loss of myocardial fibers. Groups of aged-matched uninfected wild-type (WT) and Gal-8KO were included as controls.

### Cytokine Evaluation

Cytokine levels in cardiac lysates or plasma were quantified by ELISA as we previously described (Sanmarco et al., 2016) following manufacturer's instructions (BioLegend). The total protein concentration of heart samples was determined by the Bradford method (Bio-Rad).

### Cardiac-Infiltrating Cell Isolation and Flow Cytometry

Cardiac leukocyte isolation was performed as previously described (Eberhardt et al., 2019). Briefly, hearts were perfused with phosphate-buffered saline (PBS) and disaggregated mechanically and enzymatically with 0.2% trypsin solution (Gibco). The digested tissue was pressed through a 70 μm cell strainer (BD Falcon), and cells were isolated by 35 and 70% bilayer Percoll (GE Healthcare) density gradient centrifugation. Viable cell numbers were determined by trypan blue dye exclusion using a Neubauer chamber, and absolute cell number was obtained corresponding to the whole heart. Cells were stained with the following antibodies: anti-mouse fluorescein isothiocyanate (FITC)-CD3, APC-Cy7-CD4, PE-Cy7-CD8, PE-CD19, PerCP-Cy5.5-CD11b, FITC or APC-Cy7-CD11c, PE-F4/80, PE-Cy7-CD206, APC-Ly6G, and APC-Cy7-Ly6C (all from BioLegend). Stained samples were acquired using FACS Canto I and II cytometers (Becton Dickinson), and the data were analyzed using FlowJo software (Tree Star). Non-specific fluorescence was determined using isotype controls.

### Preaparesis Assays

Circulating leukocytes were obtained by treatment of blood with lysis buffer (Gibco), stained with anti-mouse PE-CD11b and APC-Ly6G, (BioLegend), and labeled with 5 μl of FITC-Annexin V (BD Pharmingen) for 15 min on ice. Before acquisition, the cells were stained with 7-aminoactinomycin D (7-AAD) (BD Biosciences) (Stowell et al., 2008).

Stained samples were acquired using a FACS Canto II cytometer (Becton Dickinson), and data were analyzed using FlowJo software (Tree Star). For all flow cytometric procedures, an isotype control was included.

### Propidium Iodide Staining

Peripheral blood samples (30 μl) obtained from infected mice were stained with Alexa 647-Ly6G (BioLegend) and PerCP-CD11b (BioLegend). Cells were fixed with 70% ethanol for 30 min at 4°C and stained with 200 μl of propidium iodide (50 μg/ml) (Sigma).

### Tissue *Trypanosoma cruzi* Load Quantification

Genomic DNA was purified from infected heart, liver, and skeletal muscle using DNAzol reagent following the manufacturer's instructions. *T. cruzi* DNA-specific primers TCZ-forward 5′-GCTCTTGCCCACAMGGGTGC-3′, where M = A or C, and TCZ-reverse 5′-CCAAGCAGCGGATAGTTCAGG-3′, which amplifies a 182-bp product and then quantified by real-time PCR using SYBERGREEN (Applied Biosystems). Separately, reactions containing 50 ng of genomic DNA and 0.5 μM of murine-specific tumor necrosis factor (TNF) primers TNF-5241 5′-TCCCTCTCATCAGTTCTATGGCCCA-3′ and TNF-5411 5′-CAGCAAGCATCTATGCACTTAGACCCC-3′ were used as loading controls. Primer and probe sequences were described by Cummings and Tarleton (2003).

### RNA Isolation and RT-PCR

Total RNA was isolated from 50 mg of heart samples by mechanical homogenization and TRIzol (Invitrogen), as recommended by the manufacturer. The RNA was resuspended in 20 μl nuclease-free water (Epicenter) and quantified using a spectrophotometer (Nanodrop spectrophotometer ND-1000). cDNA was synthesized from 2 μg of total RNA with 0.5 μg of oligodT primers and MMLV reverse transcriptase (Promega), according to the manufacturer's instructions.

Real-time reaction was performed using Kapa SYBR® Fast qPCR kit (KapaBiosystems) in a final volume of 20 μl in a Gene Amp 7500 Sequence Detection System (Applied Biosystems). Primers used in real-time PCR assays are as follows: Gal-8: Fwd 5′-GGGTGGTGGGTGGAACTG-3′, Rev 5′-GCCTTTGAGCCCCCAATATC-3′; Gal-3: Fwd 5′-GACCACTGACGGTGCCCTAT-3′, Rev 5′-GGGGTTAAAGTGGAAGGCAA-3′ CCL2 Fwd 5′-TGCCCTAAGGTCTTCAGCAC-3′, Rev 5′-AAGGCATCACAGTCCGAGTC-3′ GAPDH Fwd 5′-ACCCAGAAGACTGTGGATGG-3′, Rev 5′-ACACATTGGGGGTAGGAACA-3′; and β-actin: Fwd 5′-CGTCATCCATGGCGAACTG-3′; Rev 5′-GCTTCTTTGCAGCTCCTTCGT-3′. Standard curves were generated for each primer set, and each PCR was run using serial dilutions of one known cDNA sample. SYBR Green data were obtained using 7500 (Applied Biosystems). Samples were analyzed by triplicate. β-Actin and GAPDH genes were used as housekeeping controls.

### Statistics

Statistical significance of comparisons of mean values was assessed by a two-tailed Student's *t*-test and two-way ANOVA followed by Bonferroni's *post-hoc* test and a Gehan–Breslow–Wilcoxon test using GraphPad software.

## Results

### Gal-8 Deficiency Favors Inflammation During *Trypanosoma cruzi* Infection

To analyze the role of Gal-8 in the context of parasite infection, we infected B6 (iWT) and Gal-8-deficient (iGal-8KO) mice with the *Trypanosoma cruzi* Ac strain that leads to chronic infection in this model (Risso et al., 2004). Parasitemia values were similar between iWT and iGal-8KO, early during the infection (data not shown). No differences in survival rate were found, and parasitemia was undetectable in both infected groups at the chronic stage (data not shown). This study was conducted at 4–5 mpi, that is, once the acute phase of the infection is solved and the chronic phase is already established.

**Figure 1:**
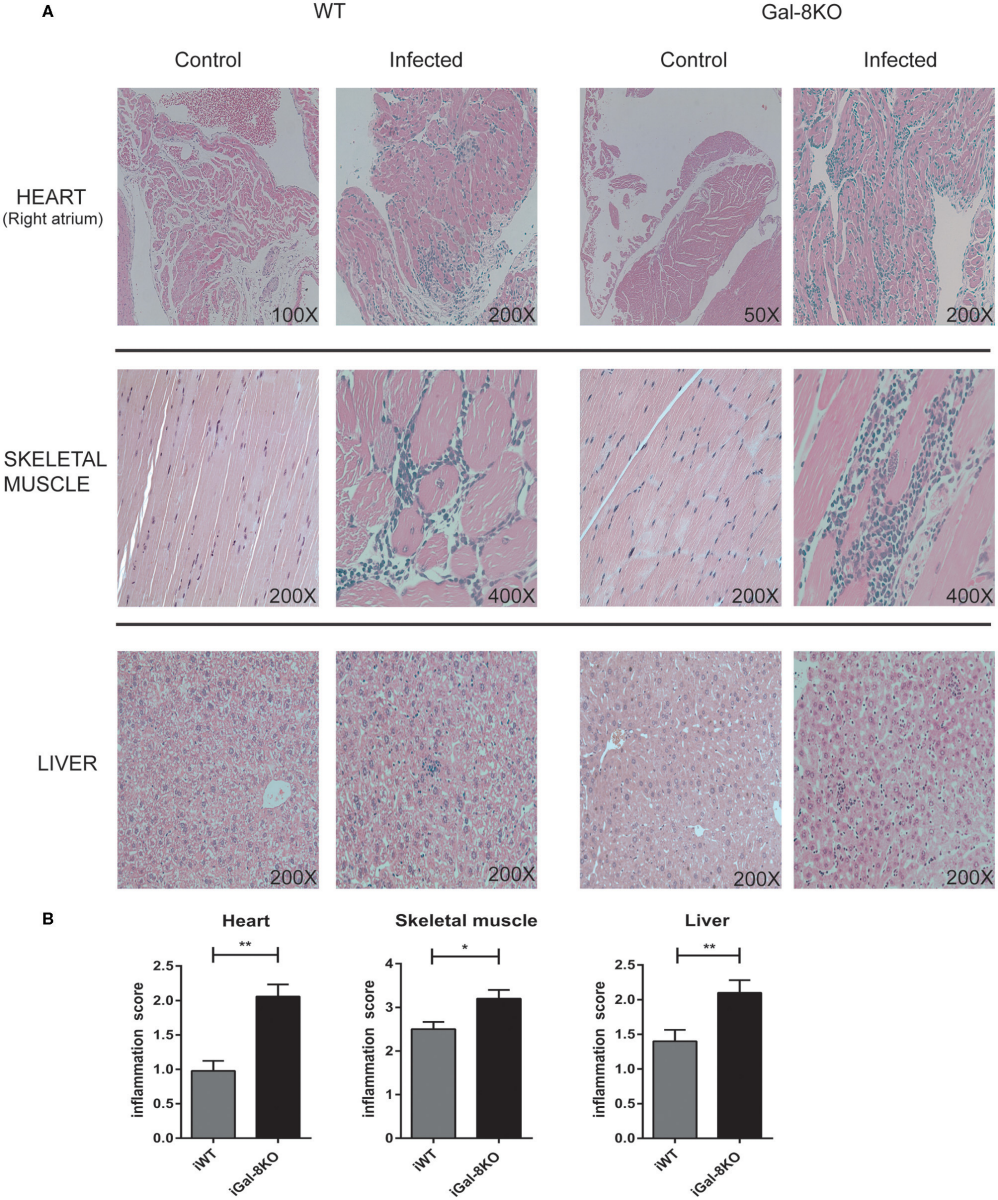
Scoring of tissue inflammation by histopathology. **(A)** Representative photomicrographs of H&E-stained sections of the heart, skeletal muscle, and liver from *Trypanosoma cruzi*-infected Gal-8KO, and wild-type (WT) mice and non-infected controls at 4–5 mpi. **(B)** Scoring of tissue inflammation was estimated as described in Methods section. Data are expressed as mean ± SEM of at least three independent experiments (5 animals/group). *P < 0.05; **P < 0.01.

**Figure 2:**
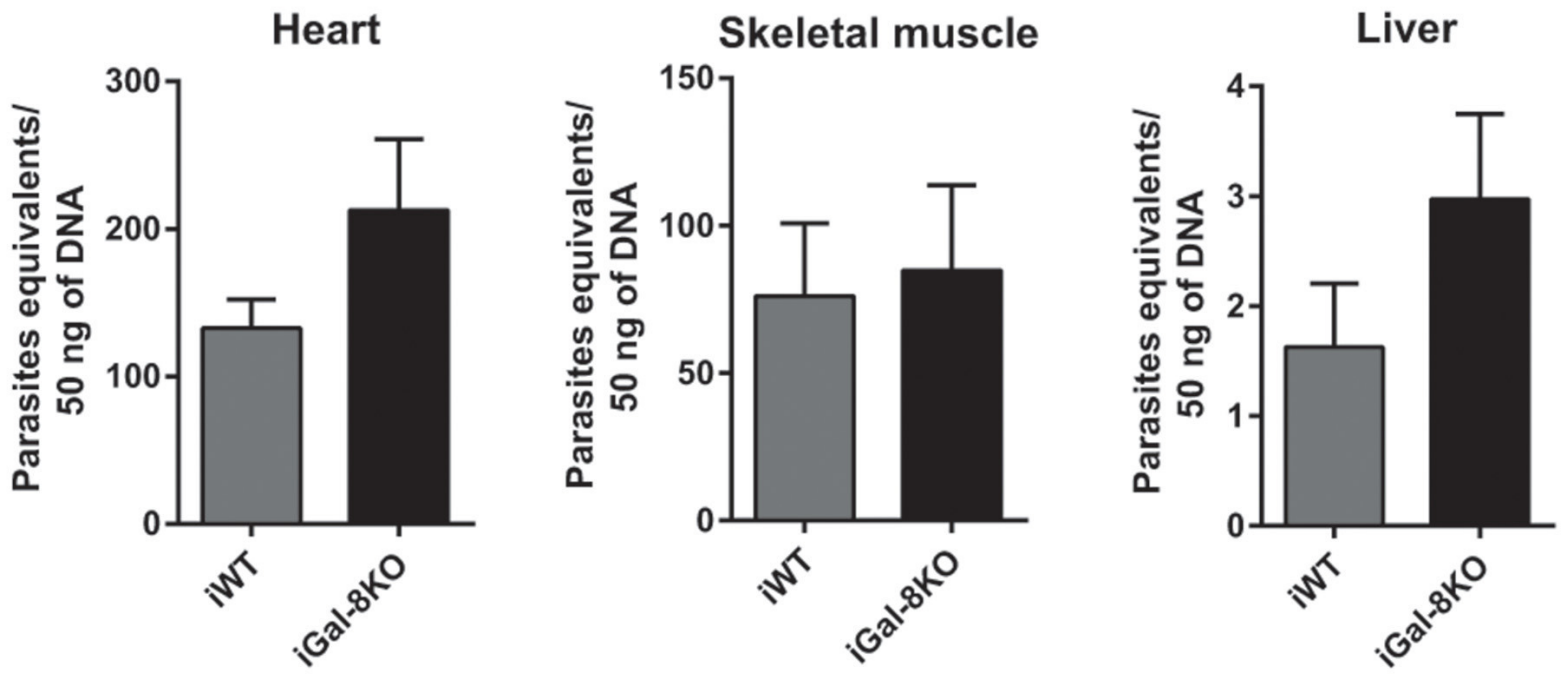
Tissue parasite burden in *Trypanosoma cruzi*-infected Gal-8KO and wild-type (WT) mice. Parasite load was determined by real-time PCR normalized to host tumor necrosis factor (TNF) in mouse tissues at 4–5 mpi. Results are expressed as mean ± SEM of parasite DNA equivalents of at least three independent experiments (5 animals/group).

To comparatively assess the inflammatory response induced by *T. cruzi* infection, cardiac tissues from iWT, iGal-8KO, and non-infected control mice were analyzed by histopathology. We observed inflammatory cell infiltrates consisting of mononuclear (lymphocytes, monocytes, macrophages, and plasmocytes) and polymorphonuclear cells only in infected tissues. A higher inflammation score was found in iGal-8KO cardiac tissue compared with iWT mice (Figures 1A,B) (*P* = 0.0014). To determine whether these findings were restricted to cardiac tissue, skeletal muscle and liver samples were also analyzed. Skeletal muscle and liver samples from iGal-8KO mice showed higher inflammatory scores than their iWT counterparts (Figures 1A,B) (*P* = 0.0150 and *P* = 0.0099, respectively). The relationship between inflammation and *T. cruzi* burden was further assessed by comparatively testing parasite load by real-time PCR in different target tissues (Figure 2). Notably, both infected groups displayed a similar parasite load in the heart, liver, and skeletal muscle (Figure 2). Altogether, these findings support that the increased inflammatory response observed in infected Gal-8 KO mice by histopathology depends mostly on the lack of a functional *Lgals8* gene in the host.

**Figure 3:**
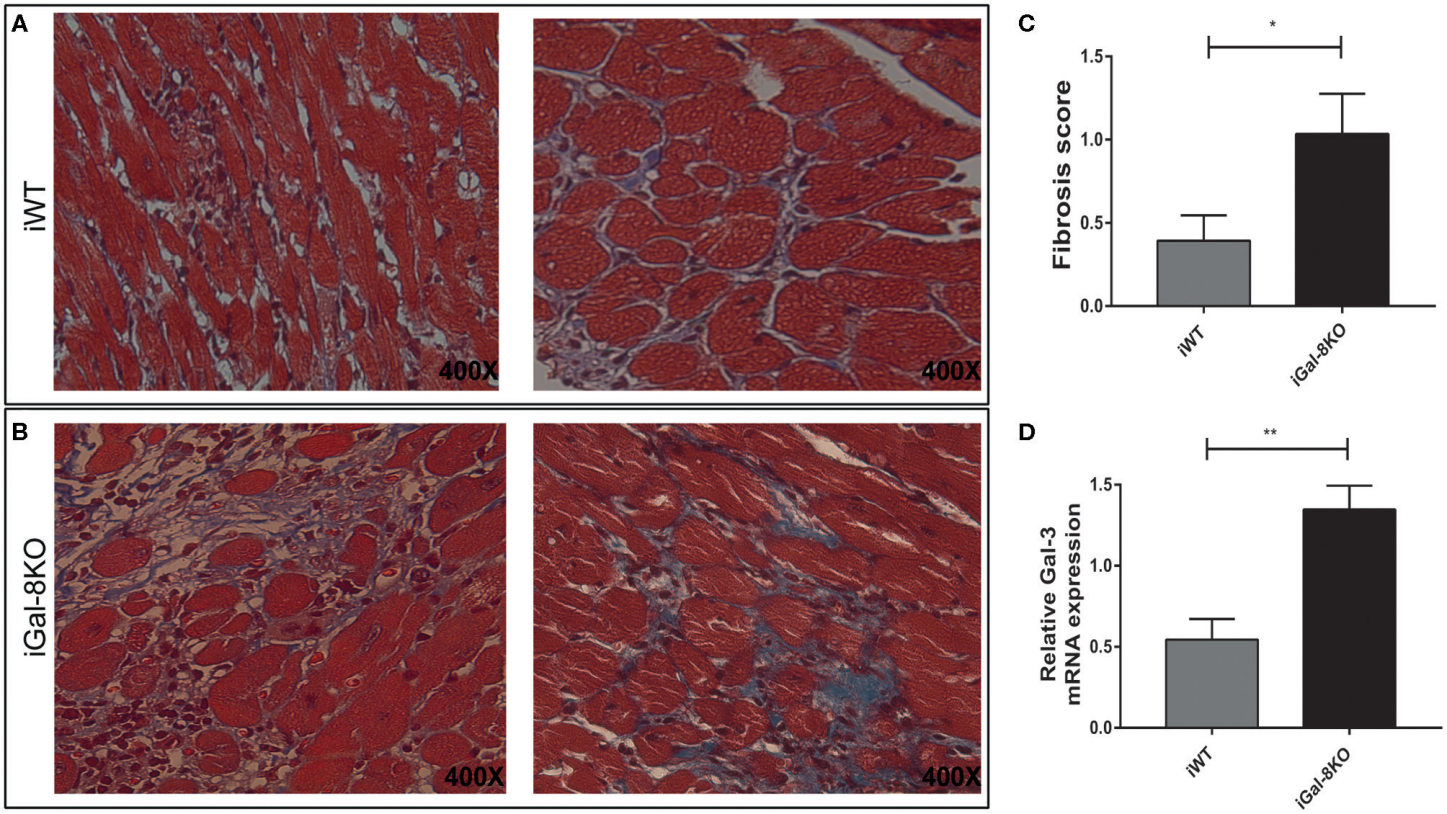
Analysis of cardiac fibrosis. Representative photomicrographs of Masson's trichrome-stained heart sections of *Trypanosoma cruzi*-infected Gal-8KO and wild-type (WT) mice at 4–5 mpi. Fibrosis severity was evaluated following the criteria described in methods section; collagenous fibers are stained blue. Note the milder fibrosis in iWT mice **(A)** and severe loss of cardiac myocytes and dense fibrous tissue surrounding individual myocardial fibers in iGal-8KO mice **(B)**. **(C)** Graphical representation of heart fibrosis scoring. **(D)** Quantification of Gal-3 mRNA expression in *T. cruzi*-infected mice heart samples by real-time PCR, normalized to host GAPDH expression. Results are expressed as mean ± SEM of at least three independent experiments (5 animals/group). *P < 0.05; **P < 0.01.

Considering that fibrosis triggered by parasite infection is a key component of cardiac remodeling, we evaluated fibrous tissue in iWT and iGal-8KO heart sections stained with Masson's trichrome. Cardiac fibrosis was more extensive and severe in iGal-8KO mice compared with the iWT mice (Figures 3A–C) (*P* = 0.044). Furthermore, the expression of cardiac Gal-3 mRNA, a molecular marker of fibrosis (Yu et al., 2013; Souza et al., 2017), was significantly higher in iGal-8KO mice compared with iWT and control mice (Figure 3D) (*P* = 0.0037). Taken together, these results suggest that cardiac remodeling follows the pattern of the inflammatory response induced by the parasite in a Gal-8-deficient scenario.

### Increased Number of Macrophages and Neutrophils Infiltrate iGal-8KO Heart

**Figure 4:**
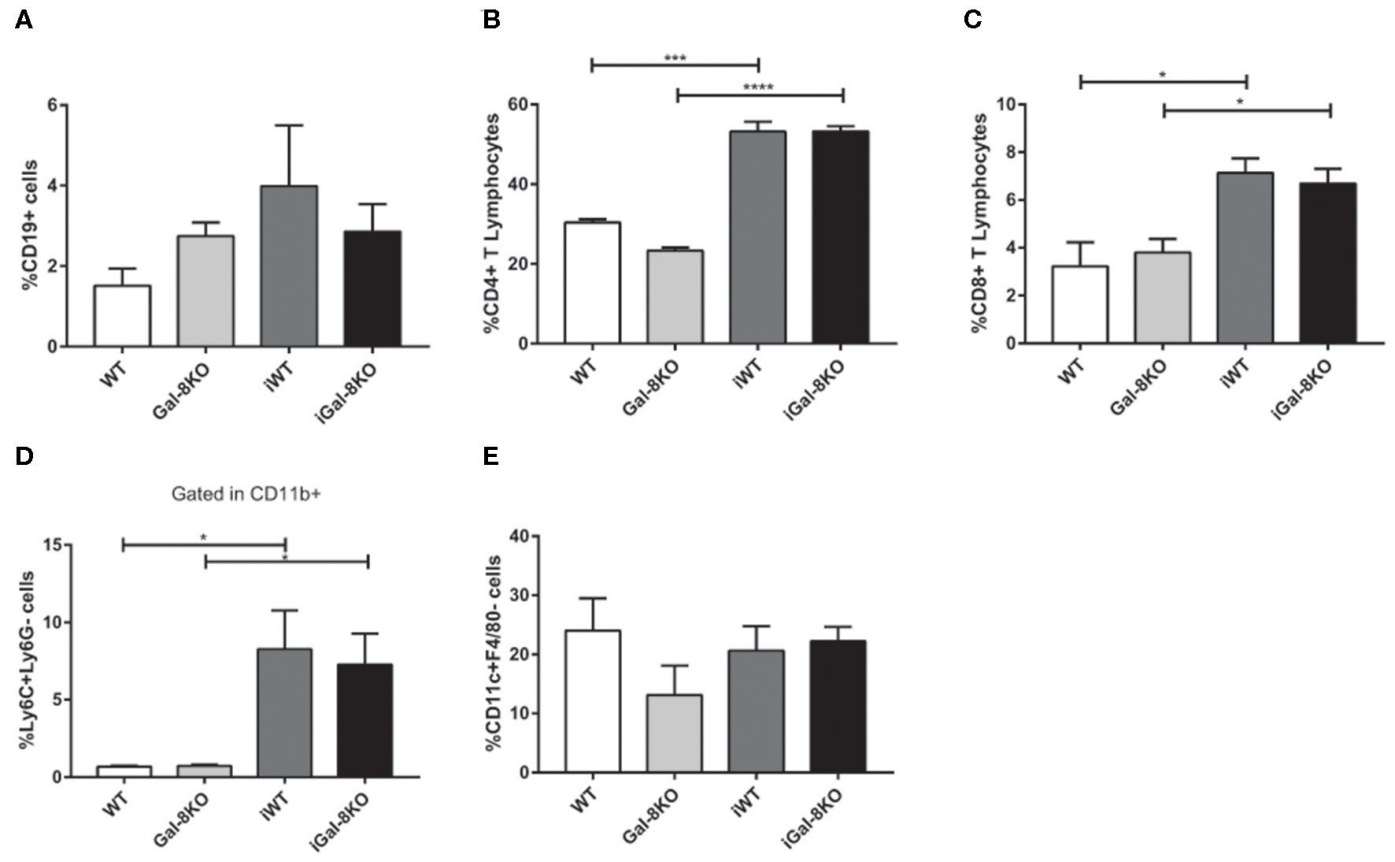
Phenotype of immune cells infiltrating chronic infected hearts. Immune cells were recovered from iWT and iGal-8KO mice heart tissues at 4–5 mpi and analyzed by flow cytometry. **(A)** Percentage of CD19+ B lymphocytes, **(B)** percentage of CD4+ T lymphocytes and **(C)** CD8+ T lymphocytes, and **(D)** percentage of CD11b+Ly6C+Ly6G– monocytes. **(E)** Percentage of CD11c+F4/80– dendritic cells. Results are expressed as mean ± SEM cells obtained in at least three independent experiments (5 animals/group). *P < 0.05; ***P < 0.001; ****P < 0.0001.

**Figure 5:**
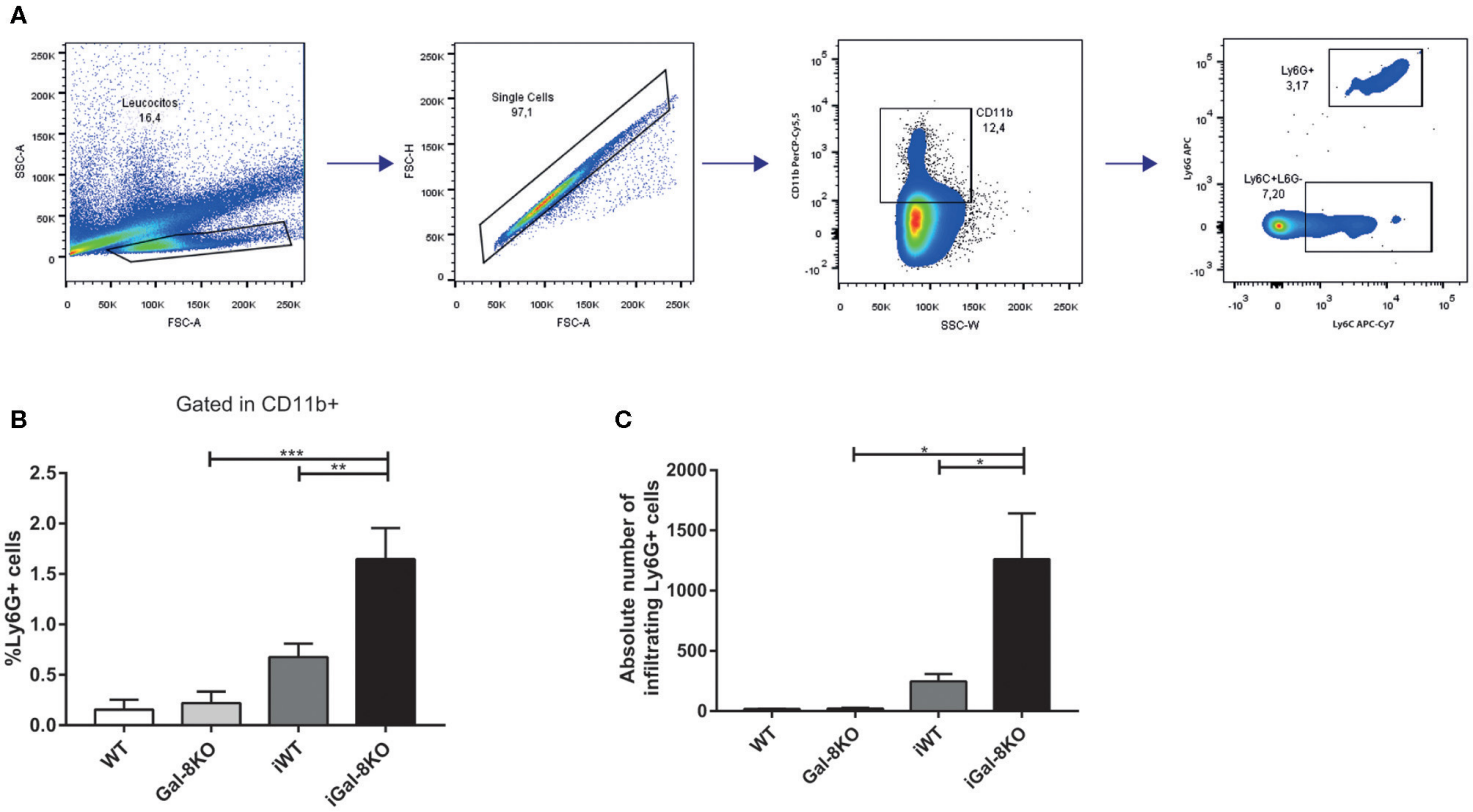
Heart-infiltrating neutrophils in infected Gal-8KO and WT mice. **(A)** Flow cytometry gating strategy for heart-infiltrating neutrophils. **(B)** Percentage of CD11b+Ly6G+Ly6C+ neutrophils. **(C)** Absolute number of cardiac neutrophils. Results are expressed as mean ± SEM cells of at least three independent experiments (5 animals/group). *P < 0.05; **P < 0.01; ***P < 0.001.

**Figure 6:**
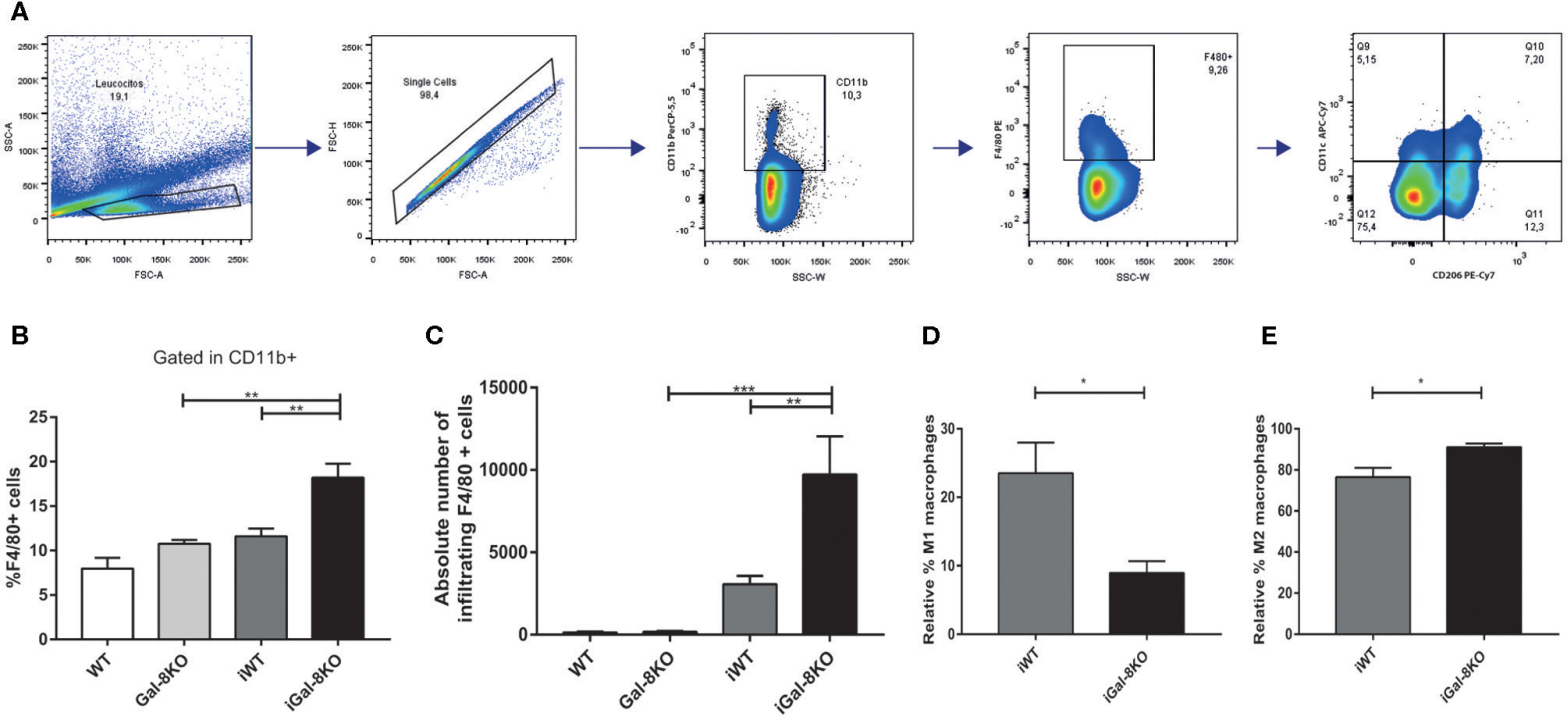
Heart-infiltrating macrophages in infected Gal-8KO and wild-type (WT) mice. **(A)** Flow cytometry gating strategy for heart-infiltrating macrophages. **(B)** Percentage of CD11b+F4/80+ macrophages. **(C)** Absolute number of cardiac macrophages. **(D)** Relative percentage of F4/80+CD11c+cd206– M1-type macrophages and **(E)** relative percentage of F4/80+CD206+CD11c+ M2-type macrophages. Results are expressed as mean ± SEM cells of at least three independent experiments (5 animals/group). *P < 0.05; **P < 0.01; ***P < 0.001.

Because inflammation is crucial for the development of *T. cruzi* cardiomyopathy, we evaluated immune cell populations infiltrating within cardiac tissues by flow cytometry. The percentage of CD3+CD4+ and CD3+CD8+ T lymphocytes and CD11b+Ly6C+Ly6G– monocytes in infected mice (iWT and iGal-8KO) were significantly increased compared with that of non-infected WT and Gal-8KO control mice (Figure 4; Supplementary Figures 1, 2) (*P* = 0.0001, *P* = 0.0053, and *P* = 0.0194, respectively). There were no significant differences in the frequency of CD19+ B lymphocytes, CD4+ and CD8+ T lymphocytes, CD11b+Ly6C+Ly6G– monocytes, and CD11c+F4/80– denditric cells between iWT and iGal-8KO hearts (Figure 4; Supplementary Figures 1, 2). In contrast, the percentage and absolute number of cardiac CD11b+Ly6G+Ly6C+ neutrophils were significantly increased in iGal-8KO compared with iWT and non-infected Gal-8KO mice (Figure 5) (*P* = 0.0104 and *P* = 0.0001, respectively). No differences were detected between naive WT and Gal-8KO mice (Figures 5B,C). The percentage and absolute number of cardiac CD11b+F4/80+ macrophages in iGal-8KO mice was also significantly higher compared with that in iWT and non-infected Gal-8KO (Figures 6A–C) (*P* = 0.0013 and *P* = 0.0003, respectively). As expected, considering the higher inflammatory rates and repair levels observed in iGal-8KO hearts, in these tissues, macrophages with M2 phenotype (F4/80+CD206+CD11c–) was predominant over M1 profile, whereas macrophages with M1 phenotype (F4/80+CD11c+CD206–) predominated over M2 profile in iWT hearts (Figures 6D,E) (*P* = 0.0225).

**Figure 7:**
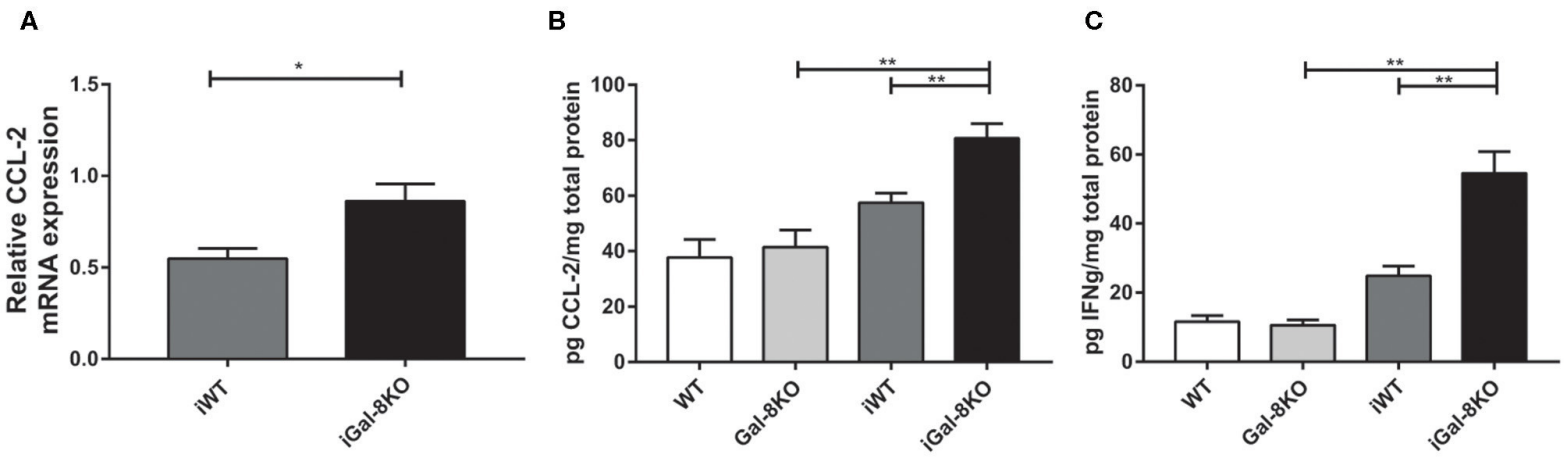
Cytokines and chemokines in hearts from infected wild-type (WT) and Gal-8KO mice. **(A)** CCL-2 mRNA quantification in infected cardiac tissues by real-time PCR normalized to host β-actin expression. Cardiac CCL-2 **(B)** and interferon γ **(C)** levels quantified by ELISA and normalized to total cardiac protein measured by the Bradford method. Results are expressed as mean ± SEM of at least three independent experiments (5 animals/group). *P < 0.05; **P < 0.01.

In accordance with these results, the expression of cardiac CCL2 levels were significantly higher in iGal-8KO than in iWT counterpart when both mRNA (*P* = 0.0166) and protein level were analyzed (Figures 7A,B) (*P* = 0.0038). Although the cardiac production of IFN-γ was also increased in the iGal-8KO group compared with iWT (Figure 7C) (*P* = 0.0006), there were no changes in other cytokines (IL-1β, IL-2, IL-4, IL-6, IL-10, IL-12, and IL-17; data not shown). Cytokine levels were similar between non-infected Gal-8KO and WT mice (data not shown).

### Analysis of Neutrophil Increment in iGal-8KO Hearts

**Figure 8:**
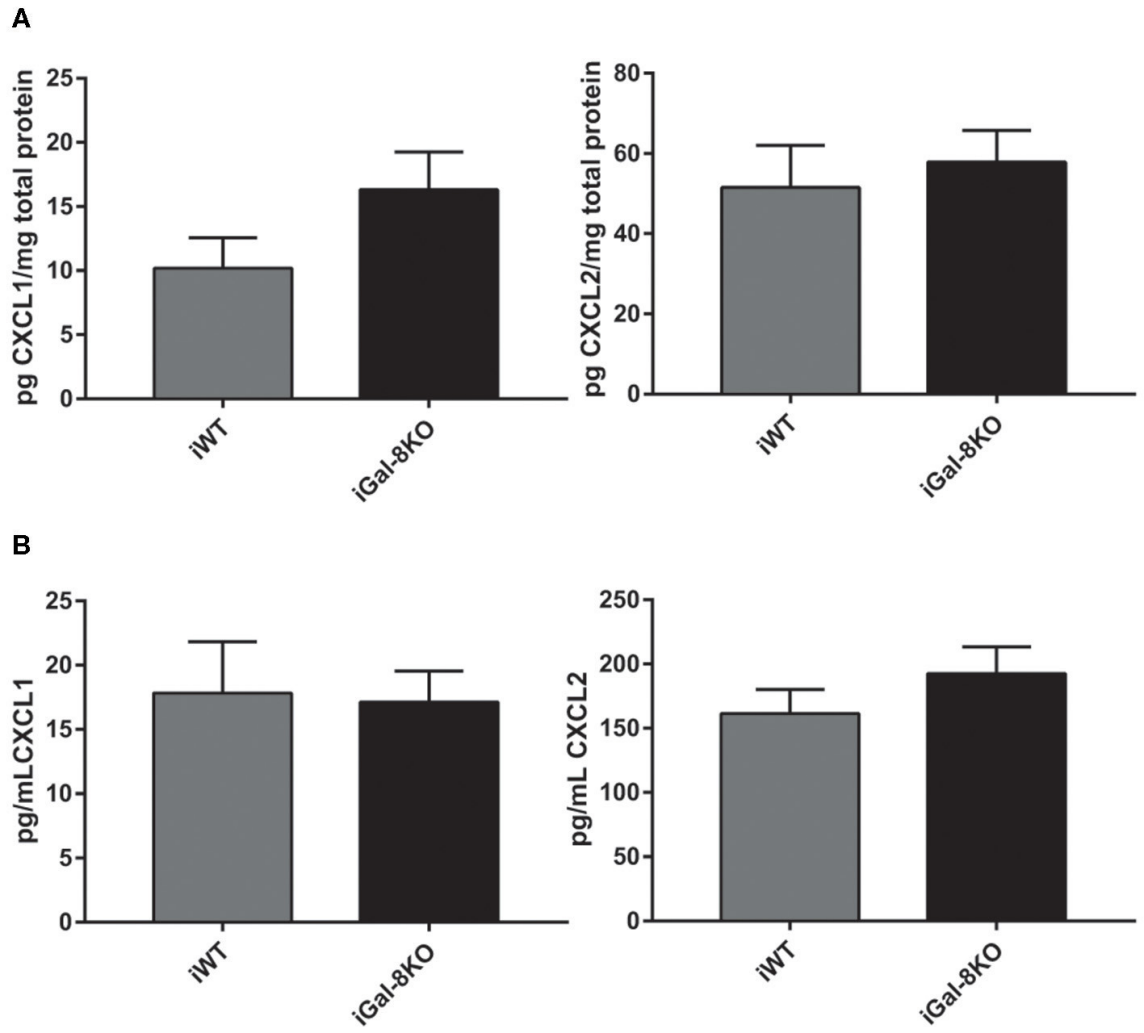
CXCL1 and CXCL2 levels in infected wild-type (WT) and Gal-8KO mice. Neutrophil chemoattractants were measured in cardiac lysates **(A)** and serum samples **(B)** by ELISA and normalized to total cardiac protein measured by the Bradford method (5 animals/group).

The increased number of neutrophils in chronic infected hearts can be a consequence of increased recruitment to the target tissue or to longer neutrophil persistence in the heart. To explore this finding, we next analyzed CXCL1 and CXCL2 chemokine levels, and we found similar values in heart and plasma samples from both iGal-8KO and iWT mice (Figures 8A,B).

**Figure 9:**
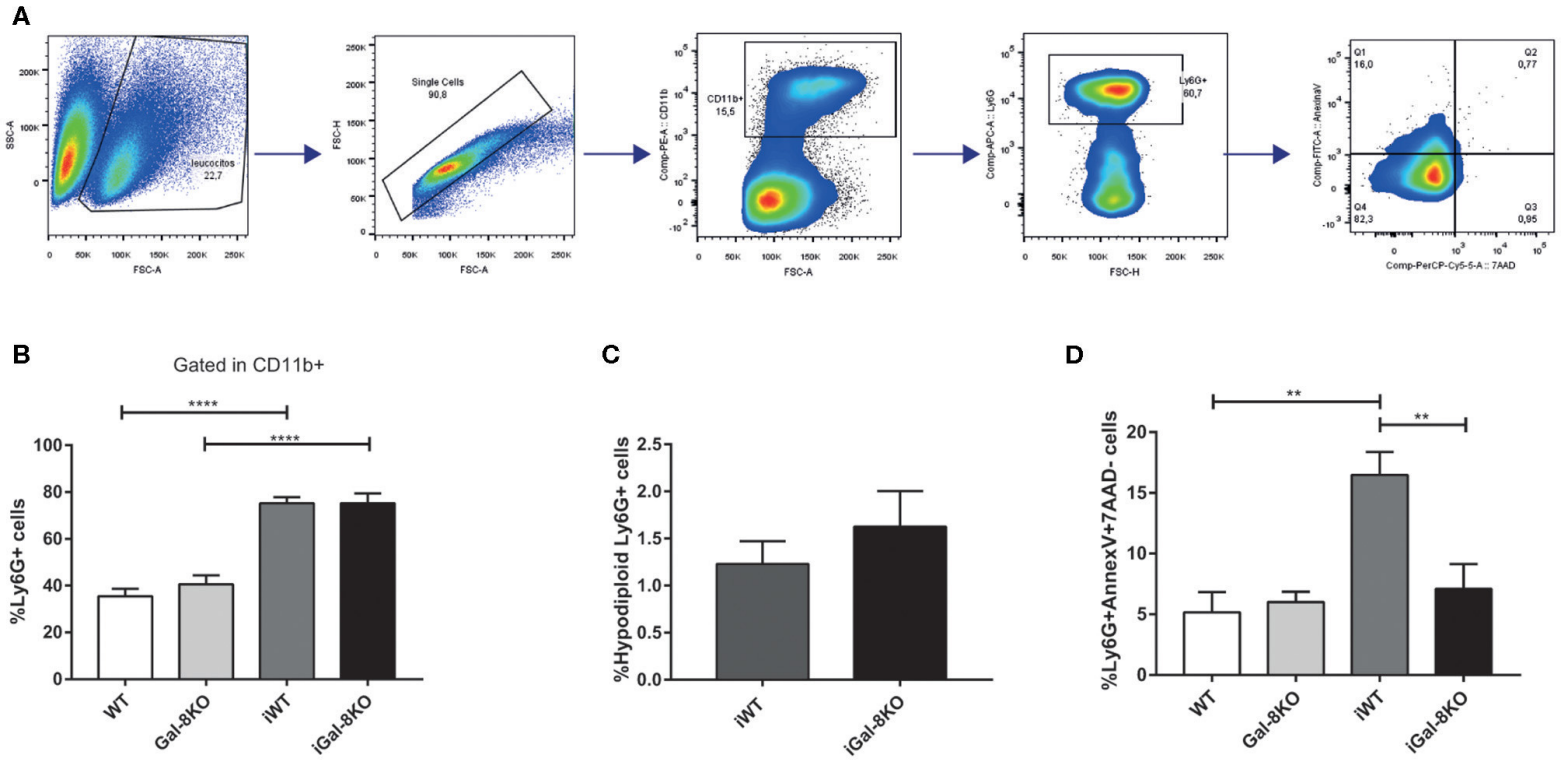
Analysis of preaparesis in peripheral blood neutrophils from *Trypanosoma cruzi*-infected Gal-8KO and wild-type (WT) mice. **(A)** Flow cytometry gating strategy for determining preaparesis in neutrophils. **(B)** Percentage of Ly6G+ neutrophils. **(C)** Measurement of hypodiploid cells. **(D)** Preaparesis was analyzed by using Annexin-V and 7-aminoactinomycin D (7-AAD) staining on Ly6G+ neutrophils. Results are expressed as mean ± SEM of at least three independent experiments (7 animals/group). **P < 0.01; ****P < 0.0001.

**Figure 10:**
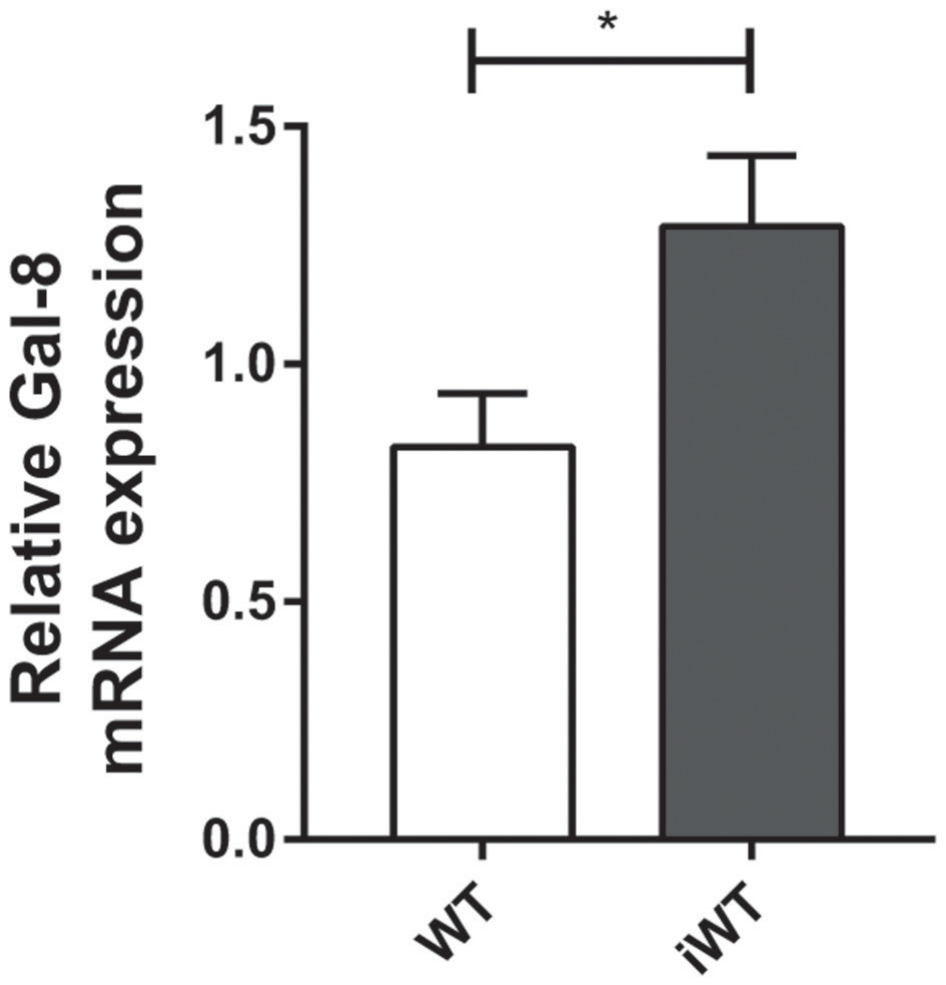
Cardiac expression of Gal-8 mRNA in infected and non-infected wild-type (WT) mice. Gal-8 mRNA was quantified by real-time PCR normalized to host GAPDH expression. Results are expressed as mean ± SEM of at least three independent experiments (5 animals/group). *P < 0.05.

Considering that Gal-8 was proposed to be involved in preaparesis (Stowell et al., 2008), we then decided to evaluate it in our experimental model. The frequency of circulating CD11b+Ly6G+ neutrophils was significantly higher in both infected group mice compared with non-infected controls (*P* = 0.0001), with no significant differences between iGal-8KO and iWT mice (Figures 9A,B). There were no differences in the apoptotic rate of CD11b+Ly6G+ cells (hypodiploid cells stained with propidium iodide) between iWT and iGal-8KO mice (Figure 9C). However, the analysis of Annexin-V expression (surface PS detection) and 7-AAD exclusion staining by viable cells showed higher frequencies of Annexin-V^+^/7-AAD^−^ cells (preaparesis-induced circulating neutrophils) in iWT than in iGal-8KO mice (Figure 9D) (*P* = 0.0001). Moreover, Gal-8 expression was increased in hearts from chronically infected WT mice (Figure 10). These results support the involvement of Gal-8 in preaparesis induction. Therefore, it is plausible to think that neutrophil accumulation can be caused by the inability of phagocytes to remove them due to the lack of an “eat-me” signal such as PS on the surface of Gal-8-deficient neutrophils.

## Discussion

The effect of the regulatory role of Gals on both innate and adaptive immunity has grown significantly recently (Blidner et al., 2015; Brinchmann et al., 2018). They are currently viewed as potential therapeutic tools for chronic inflammatory processes such as autoimmune or infectious diseases (Rabinovich and Toscano, 2009). Gal-8 has been related to the induction of platelets and cellular activation (Romaniuk et al., 2010; Cattaneo et al., 2014), co-stimulation (Tribulatti et al., 2009), and proliferation (Tribulatti et al., 2012). However, several studies have also suggested an immunosuppressor function. For instance, a pro-apoptotic role on Jurkat T-cells (Norambuena et al., 2009) and in CD4+CD8+ thymocytes has been described (Tribulatti et al., 2019). Also, the resolution of experimental autoimmune uveitis lesions is associated with Gal-8-induced T regulatory differentiation and Th17 cell exclusion (Sampson et al., 2015). It was recently reported that mice lacking Gal-8 develop autoimmune encephalomyelitis earlier and show a more severe chronic phase with increased brain inflammation (Pardo et al., 2017). Taking into consideration these precedents, we set to analyze the role of Gal-8 in an inflammatory context induced by a protozoan parasite infection. To this aim, we used the *Trypanosoma cruzi* infection, as this parasite triggers inflammatory response in different target tissues, even though it has preferential tropism for cardiac and skeletal muscles. The host's inability to eliminate the tissue parasites leads to the development of a fibrosing cardiac inflammation that alters the heart histoarchitecture and function, allowing the analysis of the role of Gal-8 along this process. Results obtained concur with the view of Gal-8 as an anti-inflammatory mediator, as its absence favors a generalized inflammatory response in different tissues (the liver, skeletal muscle, and heart) in the setting of chronic *T. cruzi* infection, with increase of neutrophils and macrophages in the heart.

In line with our findings, mice lacking Gal-1 (a well-known anti-inflammatory Gal) subjected to experimental acute myocardium infarction develop a prominent cardiac inflammatory process (Seropian et al., 2013). It was shown that during *T. cruzi* infection, Gal-1-deficient mice show increased skeletal muscle inflammation than do infected WT mice (Benatar et al., 2015). Conversely, cardiac inflammatory cells are significantly decreased in mice lacking pro-inflammatory Gal-3 under *T. cruzi* infection (Pineda et al., 2015). These reports, together with our results, let us propose that Gal-8, Gal-3, and Gal-1 are jointly involved in the modulation of cardiac inflammatory response. Intense cardiac fibrosis, evaluated by histopathology and Gal-3 mRNA expression (Ferrer et al., 2014), accompanies the inflammation level observed in both infected groups, being higher in iGal-8KO. Therefore, our findings show, for the first time, increased cardiac fibrosis in absence of Gal-8, as expected for this infection. In contrast, in *T. cruzi*-infected mice lacking Gal-3 expression (either neutralized or knocked out), fibrosis induction is highly diminished, type I collagen deposition is downregulated, and cellular proliferation is decreased (Pineda et al., 2015; Souza et al., 2017). In this sense, Gal-3 is considered a major regulator of fibrosis development (De Boer et al., 2010). Furthermore, analysis of the inflammatory infiltrate showed that the absence of Gal-8 induced a significant increase in neutrophils and macrophages in the chronic phase of the infection. In accordance with these results, levels of both monocyte chemoattractant (CCL-2) and IFN-γ inflammatory cytokine were increased. This increased production of CCL-2 by neutrophils in iGal-8KO mice hearts may be related to monocyte/macrophage population recruitment. In this sense, it has been reported that cardiac neutrophils regulate monocyte recruitment and activation in inflamed post-cardiac arrest hearts (Frangogiannis, 2018). The study of macrophage subpopulations showed that iWT hearts are enriched in M1-like population, whereas iGal-8KO hearts are predominant in the M2-like population. This predominance during a chronic inflammation and wound healing *via* fibrosis can be related to M2 macrophages' ability to synthesize ornithine, which is involved in cell proliferation and collagen biosynthesis (Wynn and Vannella, 2016). In addition, arginase activation favors *T. cruzi* persistence by promoting replication and survival (Stempin et al., 2002, 2004).

Neutrophils are known to be involved in the defense against pathogens; however, their effector mechanisms can also cause extensive damage in the surrounding tissue when uncontrolled. The elimination of apoptotic neutrophils by macrophages contributes to maintain the integrity of the affected tissue. The external exposition of phospholipids (like PS) located on the internal side of the plasmatic membrane flags the cell to be detected and engulfed by phagocytes. This process is IL-10 dependent and is considered as an anti-inflammatory pathway (Birge et al., 2016). Dr. Cummings's lab described, through *in vitro* assays, that several Gals, including Gal-8, participate in this clearing of neutrophils (Stowell et al., 2008). In this mechanism, known as preaparesis, Gal-8 induces surface expression of PS, although the cell has not entered apoptosis, and then macrophages phagocytose it *via* PS recognition. Paradoxically, in our model, the frequency of cardiac macrophages from iGal-8KO mice was increased, as was the neutrophil population. As similar levels of neutrophil chemoattractant were observed in both infected groups, this apparent incongruence could be interpreted as the host's inability to pursue the preaparesis route *via* Gal-8 to eliminate the neutrophils.

High levels of circulating viable neutrophils Annexin-V^+^/7-AAD^−^ were detected in the peripheral blood of iWT mice, which suggests that Gal-8 presence favors preaparesis induction. In contrast, neutrophils obtained from iGal-8KO mice displayed values closely similar to those observed in naive mice. Furthermore, Gal-8 expression was increased in WT hearts during chronic infection. For the first time, our findings support the development of preaparesis *in vivo* during a chronic infectious process. Even though inflammation in *T. cruzi* infection is related to parasite persistence, in our model, parasite load was similar between both infected groups in all studied tissues. Thus, parasite antigenic molecules induce an inflammatory response, but its enhancement in iGal-8KO mice can be associated with the absence of Gal-8 instead of to the tissular parasite burden, which constitutes a remarkable finding.

Neutrophils have recently been linked to diverse homeostatic or pathological events, modulating inflammation, adaptive immunity, thrombosis, atherosclerosis, and so forth (Mócsai et al., 2015). They can also adopt different phenotypes as pro-resolving or pro-inflammatory post myocardial infarction (Ma et al., 2016). In keeping with this, a study done on circulating neutrophils and monocytes from chronic Chagas patients shows a correlation between MMP-2 and IL-10 and a regulatory profile, whereas MMP-9 correlates with an inflammatory profile. Given the relevance of these cell types in the development of the cardiomyopathy, other authors have proposed them as targets for new biomarker research (Medeiros et al., 2017).

Overall, the information currently available shows how relevant this population is in the physiology of the various aforementioned processes. In this work, we present evidence that support *in vivo* neutrophil preaparesis mediated by Gal-8, as another mechanism to regulate the persistence of this innate cell type. Furthermore, this ability can be extended to other Gals that have also been involved in preaparesis induction *in vitro* (Stowell et al., 2008). Taken together, our findings show the relevance of Gal-8 involvement as an anti-inflammatory mediator in a chronic infectious disease.

## Data Availability Statement

The datasets generated for this study are available on request to the corresponding author.

## Ethics Statement

The animal study was reviewed and approved by Committee for Experimental Animal Care and Use of the Universidad Nacional de San Martín.

## Author Contributions

AB, LS, MA, and ML conceived and designed the experiments. AB, LS, and CP performed the experiments. AB, LS, CP, and MP analyzed the data. AB, LS, MP, MA, and ML wrote the paper.

## Conflict of Interest

The authors declare that the research was conducted in the absence of any commercial or financial relationships that could be construed as a potential conflict of interest.

## Supplementary Material

Supplementary Figure 1 Representative gating strategy for illustrating CD19+ B lymphocytes and CD4+ and CD8+T lymphocytes in heart tissue.

Supplementary Figure 2 Representative gating strategy for illustrating CD11c+F4/80– dendritic cells and CD11b+Ly6C+Ly6G– monocytes in heart tissue.
