# Supplementary figures and images for: Anti-inflammatory Role of Galectin-8 During Trypanosoma cruzi Chronic Infection
Source: Front Cell Infect Microbiol. 2020 Jul 2;10:285. doi: 10.3389/fcimb.2020.00285 (PMC7343849; doi:10.3389/fcimb.2020.00285)

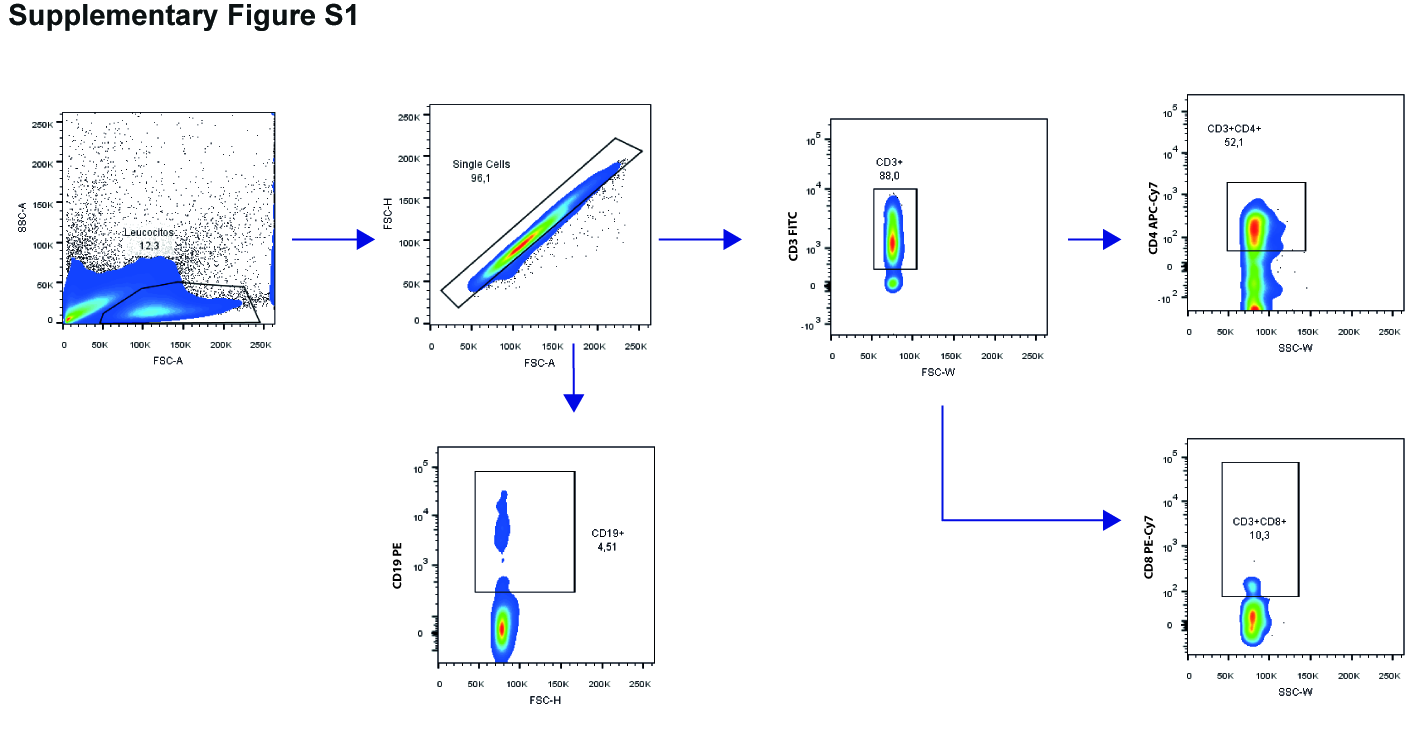

Supplement: Supplementary Figure 1 — Representative gating strategy for illustrating CD19+ B lymphocytes and CD4+ and CD8+T lymphocytes in heart tissue. [file Image_1.TIF]

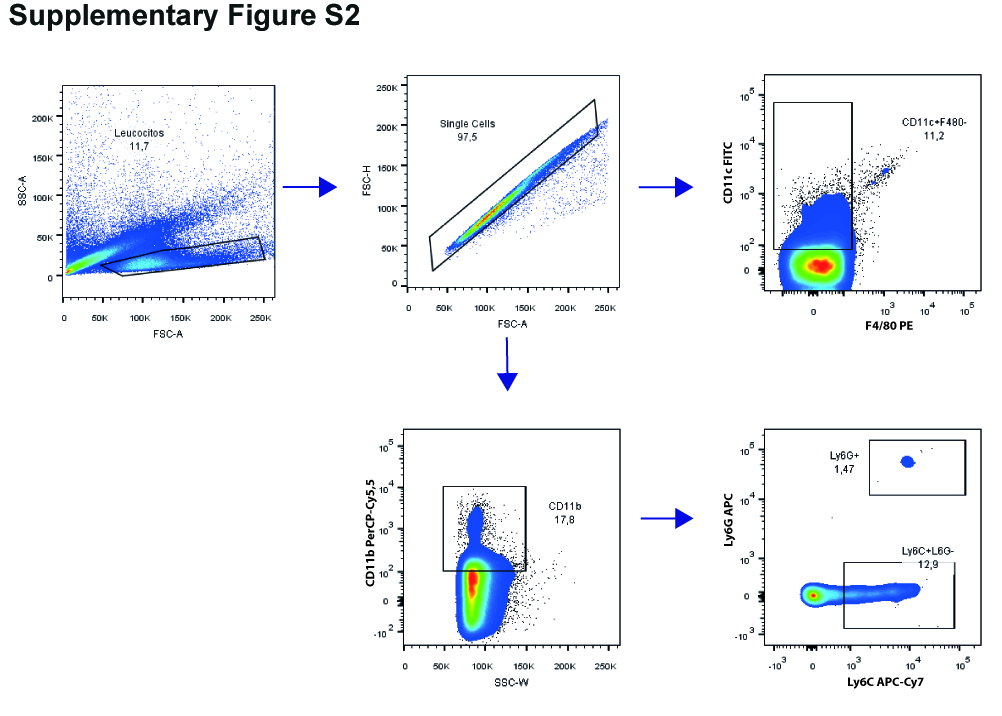

Supplement: Supplementary Figure 2 — Representative gating strategy for illustrating CD11c+F4/80– dendritic cells and CD11b+Ly6C+Ly6G– monocytes in heart tissue. [file Image_2.TIF]
